# The SIRT7-mediated deacetylation of CHD1L amplifies HIF-2α-dependent signal that drives renal cell carcinoma progression and sunitinib resistance

**DOI:** 10.1186/s13578-023-01113-4

**Published:** 2023-09-10

**Authors:** Hongchao He, Jie Li, Wei Wang, Jie Cheng, Jian Zhou, Qunyi Li, Juan Jin, Li Chen

**Affiliations:** 1 Department of Urology, Shanghai Ruijin Hospital, Shanghai Jiaotong University School of Medicine, Shanghai, 200025 China; 2 Department of Oncology, Second Affiliated Hospital, Nanjing Medical University, Nanjing, 210000 China; 3 Department of Clinical Laboratory, Lianshui County People’s Hospital, Huai’an, 223400 China; 4 Zhongshan Hospital, Fudan University, Shanghai, 200032 China; 5 Shanghai Xuhui Central Hospital, Shanghai, 200031 China; 6 Department of Pharmacy, Huashan Hospital, Fudan University, Shanghai, 200040 China; 7 Department of Nephrology, The First Affiliated Hospital of Zhejiang Chinese Medical University (Zhejiang Provincial Hospital of Traditional Chinese Medicine), Hangzhou, 310000 Zhejiang China; 8 Department of Pharmacy, Shanghai Xuhui Central Hospital, Zhongshan-Xuhui Hospital, Fudan University, Shanghai, 200031 China

**Keywords:** CHD1L, HIF-2α, SIRT7, Epigenetic reprogramming, Sunitinib

## Abstract

**Background:**

Aberrant interplay between epigenetic reprogramming and hypoxia signaling contributes to renal cell carcinoma progression and drug resistance, which is an essential hallmark. How the chromatin remodelers enhance RCC malignancy remains to be poorly understood. We aimed to elucidate the roles of CHD1L in determining hypoxia signaling activation and sunitinib resistance.

**Methods:**

The qRT-PCR, western blotting, and immunohistochemistry technologies were used to detect CHD1L expressions. Lentivirus transfection was used to generate stable CHD1L-KD cells. The roles of SIRT7/CHD1L were evaluated by CCK-8, wound healing, transwell assays, xenograft models, and tail-vein metastasis models. Co-immunoprecipitation, Chromatin Immunoprecipitation (ChIP), and luciferase reporter assays were conducted to explore epigenetic regulations.

**Results:**

We screened and validated that CHD1L is up-regulated in RCC and correlates with poorer prognosis of patients. CHD1L overexpression notably enhances cell proliferation, migration, and self-renewal capacities in vitro and in vivo. Mechanistically, SIRT7 physically interacts with CHDL1 and mediates the deacetylation of CHD1L. Wild-type SIRT7, but not H187Y dead mutant, stabilizes CHD1L protein levels via attenuating its ubiquitination levels. SIRT7 is increased in RCC and correlates with hazardous RCC clinical characteristics. SIRT7 depends on CHD1L to exert its tumor-promoting functions. Accumulated CHD1L amplifies HIF-2α-driven transcriptional programs via interacting with HIF-2α. CHD1L recruits BRD4 and increases the RNA polymerase II S2P loading. CHD1L ablation notably abolishes HIF-2α binding and subsequent transcriptional activation. CHD1L overexpression mediates the sunitinib resistance via sustaining VEGFA and targeting CHD1L reverses this effect. Specific CHD1L inhibitor (CHD1Li) shows a synergistic effect with sunitinib and strengthens its pharmaceutical effect.

**Conclusions:**

These results uncover a CHD1L-mediated epigenetic mechanism of HIF-2α activation and downstream sunitinib resistance. The SIRT7–CHD1L–HIF-2α axis is highlighted to predict RCC prognosis and endows potential targets.

**Supplementary Information:**

The online version contains supplementary material available at 10.1186/s13578-023-01113-4.

## Introduction

Renal cell carcinoma (RCC) is a common solid urological tumor, accounting for about 2.2% of adult systemic malignant cancers [1]. According to the latest statistics, there are nearly 400,000 new RCC cases each year worldwide [2]. Considering tumor heterogeneity, RCC is a spectrum of various diseases and has various histological subtypes, and genetic or molecular mutations [3]. Investigating these differences has been significant to the development of improved patient management and treatment. Clear cell renal cell carcinoma (ccRCC) is the most common subtype and accounts for 75–80% of all RCCs [4]. Previous studies have indicated that the deletion, inactivation mutation or hypermethylation of the VHL (von Hippel–Lindau) gene on the short arm of chromosome 3 is the most important carcinogenic driver for the progression of ccRCC [5]. VHL protein is an important substrate of the E3 ubiquitin ligase complex, mediating the degradation of a series of downstream proteins, including hypoxia-inducible factor (HIF), ZHX2, or SFMBT1 [6, 7]. Under pathological conditions, VHL loss-of-function often leads to accumulations of HIF proteins and stimulates the transcriptional activations of HIF downstream targets, thereby driving RCC stemness maintenance, angiogenesis, or metabolic remodeling [8]. Among these HIF subunits, HIF2α is recognized as the most important ccRCC-driven transcription factor. Therefore, a series of drugs targeting the VHL-HIF2α-VEGFA axis in RCC were investigated and developed, like PT2399, VEGFA inhibitor, or sunitinib [9, 10]. However, a majority of RCC patients exhibit resistance to these drugs in varying degrees. Abnormal HIF regulation and the activation process are important mechanisms for drug resistance [11]. Therefore, it is important to discover novel therapeutical targets or drug sensitivity predictors for RCC.

Aberrant epigenetic remodeling is involved in various stages of RCC and represents one of the most essential molecular hallmarks. Epigenetic remodeling mechanisms contain multiple aspects, like histone modification, DNA methylation, *N*6-adenosine methylation (m6A), or ubiquitination modification [12, 13]. RCC cells exploit remodeling epigenetic events to meet their nutritional needs to develop distant metastasis, proliferation, or targeted drug resistance [14]. As a result, exploring the underlying mechanisms of RCC epigenetic remodeling makes sense to discover vulnerable targets with translational significance. As is well documented, the chromatin epigenetic remodeling complex depends on ATPase activity to slide nucleosomes on the genome and control the tightness of chromatin structure, accessibility, and gene transcriptional expression [15]. Based on the structure and function of remodeling complexes, they are mainly divided into four categories: SWI/SNF complexes, ISWI complexes, INO80 complexes, and chromatin helicase-DNA binding (CHD) family [16]. We previously reported that BPTF, the largest component subunit of the ISWI complex, could enhance glycolytic activity in metastatic RCC by hijacking the super-enhancers of SRC or ENO2 [17]. On the hand, we reported that BRD9, the subunit of the SWI/SNF complex, is regulated by FTO to drive the progression of HIF2α^low/−^ RCC [18]. However, the biological roles of CHD family members in urological malignancies remain to be indefinite. CHD chromatin remodeling enzymes include nine family members, containing double chromatin domains and a central ATPase-helicase domain to exert nucleosome rearrangement and exchange functions. Among them, CHD1, CHD2, CHD3.1, and CHD4 were found to be closely related to DNA damage repair. Recently, intensive evidence demonstrated that CHD1L was highly expressed in most types of malignancy, like hepatocellular carcinoma (HCC), breast cancer, lung cancer, ovarian cancer, or colon cancer [19, 20]. CHD1L contains an SNF2-N domain and a helicase superfamily domain, implicating its roles in transcriptional regulation, maintenance of chromosome integrity, and DNA repair [21]. In liver cancer, elevated CHD1L binds to the promotor region of ZKSCAN3, one key autophagy suppressor, to inhibit its transcription. CHD1L enhances hepatocellular carcinoma by modulating the ZKSCAN3-mediated autophagy process [22]. In human non-small-cell lung cancer, overexpressed CHD1L promotes the transcription of c-Jun which is targeted directly to the promoter of ABCB1. CHD1L thus activates the ABCB1-NF-κB axis to augment cisplatin resistance of lung cancer. In this study, we also found that CHD1L potentiates the progression of RCC and mainly regulates hypoxia signaling, which deserves to be further demonstrated.

SIRT7 is one member of the sirtuin family (SIRT1–7) of mammalian NAD^+^-dependent deacetylases [23]. Unlike the other six SIRT proteins, SIRT7 is predominantly localized in the nucleus where it regulates RNA polymerase I transcription via targeting H3K18 for deacetylation [24]. SIRT7 is commonly regarded as an oncogenic driver in multiple tumors. For instance, *O*-GlcNAcylation stabilizes the SIRT7 protein to promote the aggressiveness of pancreatic cancer by blocking the SIRT7–REGγ interaction [25]. In addition, the enhanced USP17L2–SIRT7 axis modulates DNA damage response and chemo-response in breast cancer, highlighting a potential therapeutic vulnerability [26]. Nevertheless, the potential roles of SIRT7 in RCC progression are unclear now. We screened and validated that SIRT7 may regulate CHD1L proteins and relied on CHD1L to augment malignant features of RCC. However, the in-depth regulatory mechanisms of the SIRT7–CHD1L axis should be explored further.

Here, we found the oncogenic regulatory axis of the SIRT7-CHD1L axis in modulating RCC progression and sunitinib resistance. CHD1L physically interacts with HIF-2α and enhances its downstream target gene expression by increasing the recruitment of BRD4 and RNA Pol II-S2P in breast cancer cells. Targeting CHD1L via a specific inhibitor represents a novel therapeutical strategy for RCC treatment.

## Methods and materials

### Cell culture and transfection

HIF-2α^high^ (786-O, A498, Caki-1 or OSRC-2) and HIF-2α^low^ (769-P, SLR-23, Caki-2) cells were all purchased or obtained from ATCC or the laboratory of professor Li. The cell line identity was authenticated by short tandem repeat (STR) genotyping provided by the University of Arizona Genetics Core. Cells were cultured in standard DMEM or RPMI 1640 supplemented with 10% FBS. Scrambled, nontargeting siRNA was used as a negative control for transient siRNA knockdown. Lentivirus was used to establish individual stable cells, and corresponding empty vectors were used as the controls for stable cell knockdown (by shRNA) or overexpression.

### CRISPR/Cas9 genome editing

The pX459 plasmid was used to clone guide oligos targeting the CHDL1 or HIF-2α gene. In brief, 786-O or other RCC cells were plated and transfected with pX459 constructs overnight. After 24 h transfection, 1 μg/ml puromycin was used to screen cells for 3 days. Living cells were seeded in 96-well plates by limited dilution to isolate the monoclonal cell line. The knockout cell clones are screened by Western blot and validated by sanger sequencing.

### CCK-8, colony formation assay

For the CCK-8 assays, RCC cells were seeded into a 96-well plate at 3000 cells/well with 100 µl of 10% FBS DMEM. According to the protocol of CCK-8 solution (Dojindo, Kumamoto, Japan), 10 µl of CCK-8 solution diluted in 100 µl of complete culture medium replaced the original medium of each group on different days. After the cells were incubated in the dark at 37 °C for an additional 2 h, we detected viable cells by using absorbance at a 450-nm wavelength. For soft-agar colony-formation assay, cells were suspended in RPMI 1640 containing 0.35% low-melting agar (Invitrogen) and 10% FBS and seeded onto a coating of 0.7% low-melting agar in RPMI 1640 containing 10% FBS.

### Migration and invasion assays

Transwell assays were conducted in 24-well transwell plates (pore size: 8 µm; Corning, NY, USA) to assess the migratory and invasive capacities of RCC cells. For migration assays, we placed 4 × 10^4^ RCC cells in 200 µl of serum-free DMEM in the upper chamber and then added 500 µl of DMEM containing 30% FBS to the lower chamber. For the invasion assays, we precoated the chamber inserts with 50 µl of 1:6 mixture of Matrigel (BD Biosciences) and DMEM for about 2 h in a 37 °C incubator. Then we seeded 8 × 10^4^ RCC cells in the upper chamber. The lower chamber also had 500 µl of DMEM containing 30% FBS. After the cells were incubated for 48 h, we used 4% paraformaldehyde to fix the cells that had migrated or invaded the lower surface of the membrane. The crystal violet was applied to staining the fixed cells for 15 min. Five random 100× microscopic fields were selected to count the stained cells by using an IX71 inverted microscope (Olympus Corporation). We repeated all of our assays three times in our study.

### Chromatin immunoprecipitation (ChIP)-qPCR assay

The 786-O Cells (5 × 10^6^) were cross-linked followed by the preparation of nuclear lysates using Magna ChIPTM protein G Kit (Millipore, Burlington, MA, USA). Nuclear lysates were sonicated to shear DNA to around 500 bp followed by immunoprecipitation for 16 h at 4 °C using IgG or anti-CHD1L or anti-HIF-2α antibody (Genetex, San Antonio, TX, USA). The levels of targeted genes in ChIP products were determined by RT-qPCR.

### Luciferase reporter assay

The 786-O cells were plated on 48-well plates and transiently transfected with HIF luciferase reporter plasmid p2.1 containing a HRE (5ʹ-ACGTG-3ʹ) from the human VEGFA gene. The control reporter plasmid pSV-Renilla; shSC, shCHD1L#1, shCHD1L#2, pcFUGW-3xFLAG-CHD1L, pcDNA3.1-MBD3, pcDNA3.1-MTA2, or empty vector (EV). 24 h later, cells were exposed to 20% or 1% O_2_ for 24 h. The firefly and Renilla luciferase activities were measured using the Dual-Luciferase Reporter Assay System (Promega).

### Western blotting and immunoprecipitation

Protein extracts for western blotting were prepared in Laemmli loading buffer (0.1 M Tris–HCl (pH 7.0), 4% SDS, 20% glycerol, 1 mM DTT, and protease inhibitors), then separated by SDSpolyacrylamide gels, transferred to PVDF membrane (Millipore) and probed with respective antibodies. Immunoblots were visualized by the Bio-Rad system. For immunoprecipitation, cells with indicated treatments were lysed in 200 mM KCl, 20 mM Tris–HCl (pH 7.9), 5 mM MgCl2, 10% glycerol, 0.2 mM EDTA, and 0.1% NP-40, supplemented with protease inhibitors (Roche Complete). Clear cell lysates were then incubated with the respective antibodies or control IgGs at 4 °C overnight. Beads-bound immunoprecipitates were washed, eluted in Laemmli loading buffer, and analyzed by western blotting. The antibodies in this study were listed as the following: anti-SIRT7 (abcam, ab259968), anti-CHD1L (abcam, ab197019), anti-HIF-2α (CST, #87179), anti-BRD4 (abcam, ab243862), anti-Flag (abcam, ab205606), anti-HA (abcam, ab9110), anti-GAPDH (abcam, ab8245). The uncropped WB graphs were shown in Additional file 1: Figure S1.

### In vitro deacetylation assay

Briefly, SIRT7-KD 786-O cells were pre-treated with HDAC inhibitors (10 mM NAM, 50 nM TSA, 5 mM Sodium butyrate) for 6 h, then lysed in 300 mM KCl, 20 mM Tris–HCl (pH 7.9), 5 mM MgCl2, 0.2 mM EDTA, 10% glycerol, 0.5 mM DTT supplemented with 0.1% NP-40, protease inhibitors (Roche) and HDAC inhibitors (Sigma). CHD1L was purified and enriched. The acetylation level of CHD1L was monitored by western blotting with anti-pan-acetylation lysine antibodies.

### Animal studies

Pathogen-free male BALB/c and athymic nude mice were purchased from the Slaccas (Shanghai). All mice were housed and handled by protocols approved by the Committee of Ruijin Hospital, Shanghai Jiaotong university. We did preliminary experiments to determine the need for a mice sample size. All mice were assigned randomly to experimental groups and we did not perform blindly. 786-O cells (1 × 10^6^) were injected into the lateral tail vein of athymic nude mice. The number of metastatic nodules on the surface of the lung was counted under dissecting microscope after H&E staining. For sunitinib or CHD1Li treatment, BALB/c mice were inoculated with 5 × 10^5^ 786-O cells in the kidney. About 7 days after inoculation, tumor-bearing mice were randomized for treatment with vehicle, sunitinib or CHD1Li every other day and killed on day 28 for lung metastasis examination. Tumor diameters and mouse weight were monitored four times weekly. Tumor volume (mm^3^) was calculated by the formula:

$$ {\text{Volume}} = 0.5 \times {\text{length}} \times {\text{width}}^{2} . $$

### Statistical analysis

All experiments were carried out with at least three replicates. The data were shown as mean ± S.D. or mean ± S.E.M. as indicated in the figure legends. For comparison of central tendencies, normally distributed data sets were analyzed by unpaired two-sided Student’s t-tests under assumption of equal variance. Non-normally distributed data sets were analyzed by non-parametric Mann–Whitney U-tests. χ^2^-test was applied to analyze the relationship between SIRT7 levels and pathological status. Differences were considered as statistically significant when *P* < 0.05.

## Results

### CHD1L is up-regulated in RCC and associated with poor prognosis

**Fig. 1:**
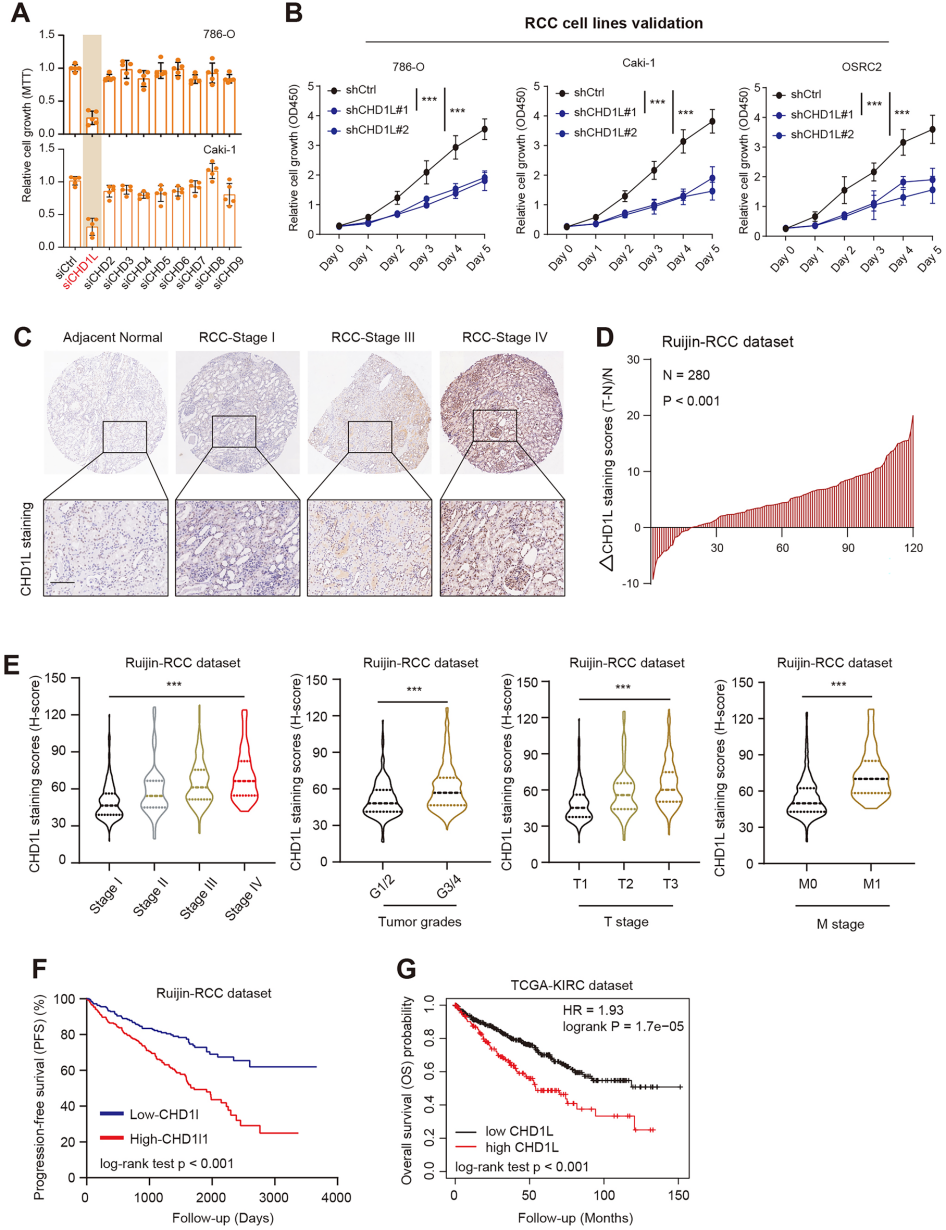
High CHD1L correlated with poorer prognosis of RCC patients. **A** siRNA KD of 9 candidate CHD family members and their effects on the growth of RCC (786-O or Caki-1) cells. The quantitative results shown are representative of 5 experiments. **B** CCK-8 assays revealed the growth rates of RCC cells transfected with shCtrl or shCHD1L#1/2 lentiviruses, individually. **C** Representative immunohistochemistry (IHC) staining graphs of CHD1L in normal or RCC samples with different tumor grades. **D** The distribution of the difference in CHD1L immunoreactivity score (△scores = (Tumor − Normal)/Normal). The CHD1L staining scores were quantified in 120 pairs of tumors from the Ruijin-RCC dataset. **E** Correlation analysis was conducted via the Kruskal–Wallis test to confirm the relationships between CHD1L h-scores and hazard clinical characteristics. **F**, **G** Kaplan–Meier survival curve analysis was conducted to compare the prognostic differences between CHD1L-high and CHD1L-low patients in Ruijin-RCC or TCGA-KIRC datasets. *p < 0.05, **p < 0.01, ***p < 0.001, ns no significant

To explore the potential roles of all CHD family members (CHD1-CHD9) in RCC, we performed a low throughput screen using individual siRNAs to evaluate their effects in RCC (786-O, Caki-1) cells (Fig. 1A). As indicated by the MTT assay, CHD1L is most potent candidate compared with others, and CHD1L ablation notably resulted in a significant decrease in cell growth. In addition, we obtained the shRNAs to target CHD1L and CHD1L knockdown remarkably reduced the proliferation rate of three RCC cell lines as evaluated by the total cell viability over a period of five days (Fig. 1B). We further obtained a panel of RCC micro-array containing 280 cases and performed immunohistochemistry (IHC) to determine the CHD1L levels. As expected, CHD1L is not only up-regulated in RCC samples versus adjacent normal sections but associated with tumor clinical stages (Fig. 1C, D). Based on the Ruijin-RCC dataset, Kruskal–Wallis (K–W) test analysis further confirmed that CHD1L expressions correlated positively with advanced clinicopathological stages, tumor grades, T stages, as well as metastatic status (Fig. 1E). Lastly, we categorized the RCC cases into CHD1L-high and -low groups according to the h-scores. Kaplan–Meier curve analysis indicated that patients with high CHD1L have poorer prognosis as compared to those with low CHD1L samples (N = 280, log-rank test *P* < 0.001, Fig. 1F). We further queried the expression data of CHD1L from the TCGA-KIRC dataset and obtained the coincident findings, in which CHD1L-high patients have shorter OS months relative to CHD1L-low samples (N = 530, log-rank test *P* < 0.001, Fig. 1G, Additional file 2: Table S1). Collectively, these data implicated that the expression level of CHD1L is significantly increased in RCC and is associated with the prognosis of patients.

### CHD1L maintains the growth and aggressiveness of RCC cells in vitro and in vivo

**Fig. 2:**
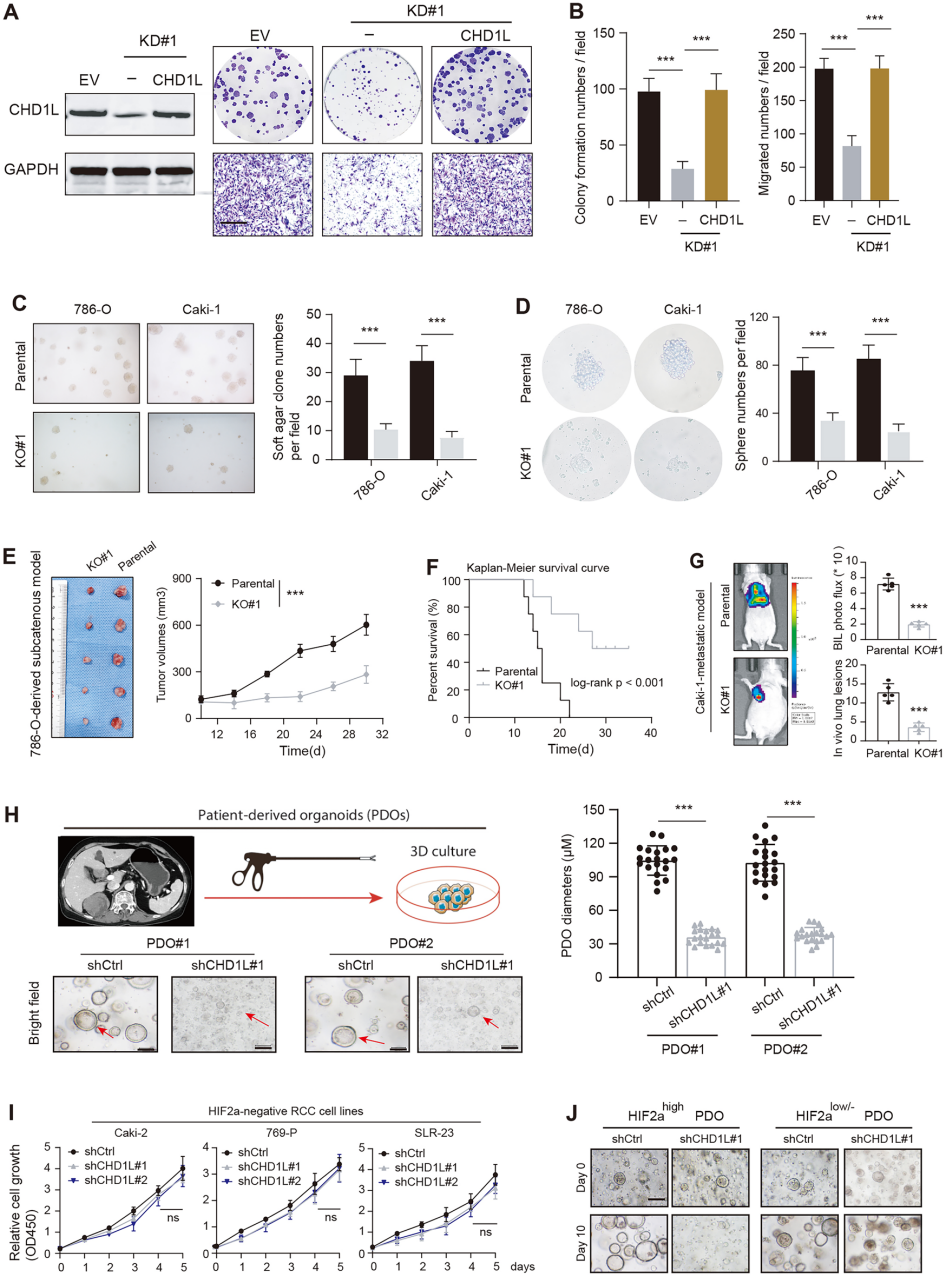
CHD1L enhances RCC malignant progression in vitro and in vivo. **A** Colony formation and Transwell (right) assays of CHD1L-KD 786-O cells with or without WT CHD1L restoration. The western blot assay showing CHD1L proteins was exhibited on the left side. **B** Quantification of colony or migration numbers in the indicated groups. **C** Soft agar colony formation showed the numbers in control or CHD1L-deficient 786-O cells. **D** Sphere formation assay revealed the stemness features of parental or CHD1L-deficient 786-O cells. **E** Measurement of subcutaneous tumor growth of control and CHD1L-deficient 786-O cells (2-way ANOVA followed by Tukey’s multiple comparisons tests), scale bar = 1 cm. **F** Kaplan–Meier analysis was used to compare the survival differences in the indicated groups. **G** Representative bioluminescence graphs showed the metastatic signals in mice injected with control or CHD1L-KO 786-O cells, individually. **H** Generation of RCC patient-derived organoids (PDOs) and representative growth images in the PDOs transfected with shCtrl or shCHD1L lentiviruses. Quantification of PDO diameters in the indicated groups. Scale bar = 100 μm. **I** CCK-8 assays showed the cell growth rates in HIF-2α^−/low^ RCC cells transfected with shCtrl or shCHD1L lentiviruses. **J** Representative graphs of HIF2^high^ and HIF2^low/−^ ccRCC PDOs treated with shCtrl or shCHD1L#1 viruses for 10 days. Scale bar = 100 μm. *p < 0.05, **p < 0.01, ***p < 0.001, ns no significant

To determine whether CHD1L is functionally required for RCC cells, we constructed the stable CHD1L-KD cell lines via shRNAs. We observed that CHD1L knockdown notably attenuated the growth and migration of 786-O cells, but the ectopic expression of CHD1L could restore the impaired oncogenic capacities of CHD1L-deficient cells (Fig. 2A, B). The soft-agar colony formation assay further confirmed that CHD1L ablation could suppress clonogenicity (Fig. 2C). The sphere formation abilities of 786-O or Caki-2 cells were notably impaired caused of CHD1L loss (Fig. 2D). To further investigate the biological role of CHD1L in vivo, we utilized a subcutaneous xenotransplantation assay to explore whether CHD1L contributed to in vivo RCC development. As compared to control tumors derived from shCtrl cells, tumors derived from CHD1L-ablated cells showed a lower tumor growth rate (Fig. 2E). Meanwhile, mice derived from CHD1L-KO group have a more favorable prognosis relative to control mice (Fig. 2F). To explore the function of CHD1L in RCC in vivo lung metastasis, we constructed the mouse model via intravenous injection of 786-*O*-luc-parental, or 786-*O*-luc-CHD1L-KO#1 cells (1 × 10^6^) into the tail vein. The Bioluminescence (BIL) imaging or lung metastatic lesions all proved that CHD1L ablation could attenuate the distal metastatic burden of RCC (Fig. 2G). Considering that organoids could structurally and functionally simulate real organs and reflect drug sensitivity, we thus constructed the patient-derived organoids (PDOs) using fresh RCC samples. The lentivirus-mediated CHD1L knockdown could notably decrease RCC-PDO growth rates, as compared to PDOs infected with shCtrl viruses (Fig. 2H). Intriguingly, we also detected the role of CHD1L in HIF-2α^low/−^ RCC, and CHD1L knockdown could mildly infect the growth rate of these cells (Caki-2, 769-P, SLR-23) (Fig. 2I). Last of all, CHD1L was knocked down in PDOs derived from HIF-2α^high^ or HIF-2α^low/−^ RCC samples, individually. We could observe that HIF-2α^high^ PDOs showed more sensitive to CHD1L depletion than those of PDOs with low HIF-2 activity (Fig. 2J). Therefore, our data suggested that CHD1L is indispensable for HIF-2α-positive RCC to sustain tumor growth and metastasis, but not the HIF-2α^low/−^ subtype.

### SIRT7 deacetylates and stabilizes CHD1L proteins via attenuating ubiquitination levels

**Fig. 3:**
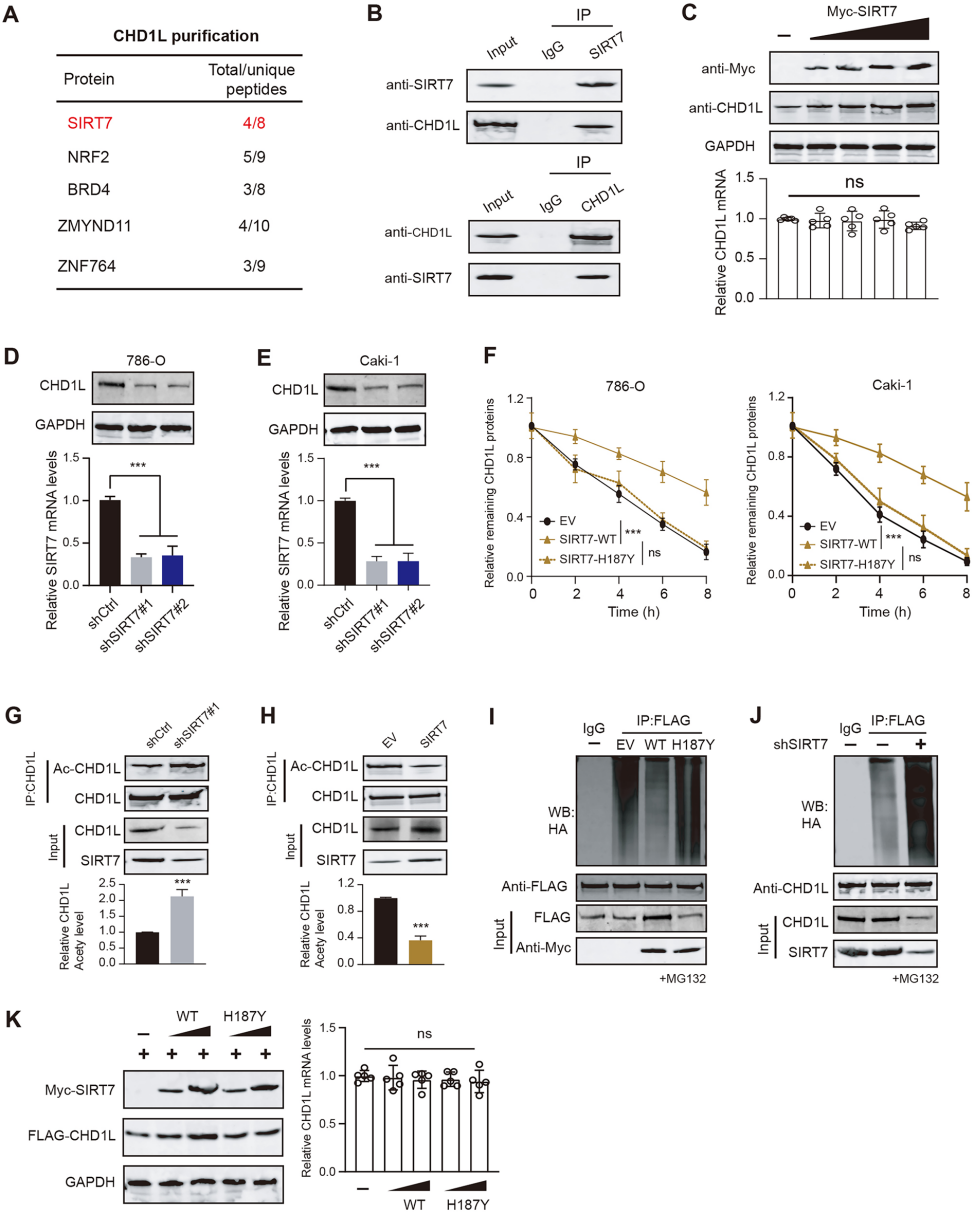
SIRT7 deacetylates CHD1L to stabilize its proteins. **A** Tandem affinity purification of CHD1L-containing protein complex was conducted using 786-O cells stably expressing double tag Flag-HA-CHD1L. Mass spectrometry was used to analyze the peptides. **B** Co-immunoprecipitation (Co-IP) of endogenous CHD1L with anti-SIRT7 antibodies (upper) and endogenous SIRT7 with anti-CHD1L antibodies (lower) in 786-O cells. Western blot was used to show the results. **C** Western blotting assay showing the CHD1L proteins (upper) and mRNA (lower) in cells transfected with increasing Myc-SIRT7 plasmids. **D**, **E** Western blotting assay detected the SIRT7 expressions in control and SIRT7-KD cells. **F** Western blot of CHD1L proteins in WCLs of 786-O or Caki-1 cells transfected with the indicated SIRT7 plasmids for 48 h and then treated with CHX (50 μg/ml) and harvested at different time points. **G** Acetylation level of CHDL1 in 786-O cells treated with shCtrl or shSIRT7 lentiviruses probed with pan anti acetyl lysine antibodies. Lower is quantitative data showing an increased level of CHDL1 acetylation in cells with SIRT7 KD. **H** Acetylation levels of CHDL1 in control or SIRT7-OE cells. Lower is quantitative data showing an increased level of CHDL1 acetylation. **I** Flag-CHD1L, Myc-SIRT7, and HA-Ub were co-transfected into 786-O cells. Immunoblots with anti-HA antibody showing polyubiquitination of CHD1L in the presence or absence of ectopic SIRT7 or H187Y mutant. Cells were treated with MG132 (15 µM) for 12 h. **J** Immunoblots with anti-HA antibody showing polyubiquitination of CHD1L in control or SIRT7-KD cells. Cells were treated with MG132 (15 µM) for 12 h. **K** Immunoblots showing CHD1L proteins in cells transfected with increasing doses of Myc-SIRT7 or H187Y plasmids. The CHD1L mRNA levels in the indicated groups were shown on the right. *p < 0.05, **p < 0.01, ***p < 0.001, ns no significant

To investigate the upstream mechanisms that contribute to aberrant CHD1L levels, we generated the stable CHD1L-overexpressing 786-O cells via transfecting the Flag-HA-CHD1L plasmids. Tandem Affinity Purification (TAP) process was performed and the CHD1L proteins were enriched. Mass Spectrometry (MS) technology was used to detect the potential CHD1L-binding peptides and SIRT7 was notably highlighted (Fig. 3A). Given that SIRT7 is intensively reported to impact tumorigenesis of multiple tumors, we thus wondered whether SIRT7 could regulate CHD1L to enhance RCC progression. To test this possibility, we first conducted co-immunoprecipitation (Co-IP) assay in 786-O cells, and CHD1L and SIRT7 was observed to have endogenous interactions (Fig. 3B). Besides, we transfected the 786-O cells with increasing amounts of Myc-SIRT7 plasmids and we observed the consistent increase of CHD1L protein expressions (Fig. 3C). However, the mRNA levels of CHD1L was not altered in response to elevated SIRT7 levels, implicating that SIRT7 may manipulate the post-transcriptional modifications (PTMs) of CHD1L proteins (Fig. 3C). Accordingly, SIRT7 KD could notably decrease CHD1L proteins (Fig. 3D). The actinomycin D assay was further used to detect the half-time of CHD1L proteins, in which overexpression of SIRT7 could largely prolong the half-life of CHD1L protein in 786-O or Caki-1 cells. The enzyme-dead SIRT7 mutant (SIRT7–H187Y) was also transfected into RCC cells, and we found that defective SIRT7 mutant failed to stabilize CHD1L proteins (Fig. 3F). We then explored whether SIRT7 deacylase activity modulates SIRT7 proteins. Expectedly, the anti-pan-acetyl K antibodies were used to detect the acetyl lysine (K) levels of CHD1L, which were remarkably elevated in SIRT7-KD 786-O cells (Fig. 3G). In contrast, SIRT7 overexpression could reduce the acetyl lysine (K) levels of CHD1L (Fig. 3H). As is well known, acetylation and ubiquitination often influence mutually to modulate stability of proteins. We thus asked whether aberrant deacetylation of SIRT7 could impact the ubiquitination process of SIRT7. Indeed, wild type SIRT7, but not the enzyme-dead mutant (H187Y), could attenuate the ubiquitination levels of CHD1L (Fig. 3I). In contrast, SIRT7 KD notably increased the ubiquitination levels of CHD1L (Fig. 3J). We co-transfected the 786-O cells with increasing doses of Flag-CHD1L and Myc-SIRT7 or its mutant (H187Y). In line with our expectations, only wild-type SIRT7 could gradually elevate CHD1L proteins, but not the H187Y mutant. Neither wild-type SIRT7 nor H187Y mutant could impact CHD1L mRNA levels (Fig. 3K). Together, SIRT7 mediates the deacetylation of CHD1L proteins and stabilizes it via attenuating the ubiquitination process.

### SIRT7 relied on CHD1L to promote RCC malignant features

**Fig. 4:**
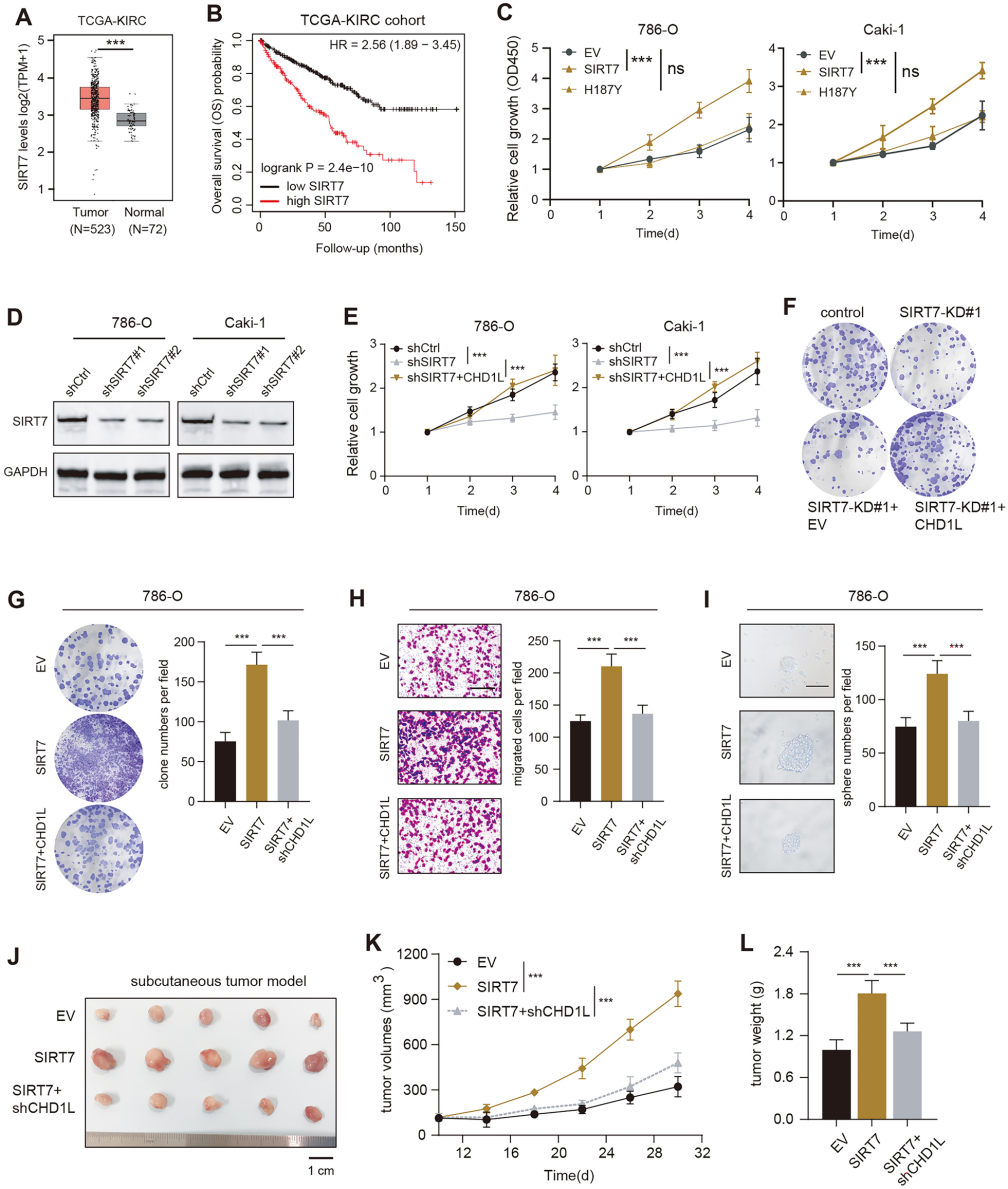
Oncogenic SIRT7 depends on CHD1L to augment RCC malignant aggressiveness. **A** Boxplot showing the differential expression levels of SIRT7 in normal and tumor samples from TCGA-KIRC. **B** Kaplan–Meier survival curves comparing the differential prognosis of SIRT7-high or -low KIRC patients. **C** CCK-8 assays show the growth rates in cells transfected with EV, wild-type SIRT7, or H187Y plasmids. **D** Western blotting assays reveal the protein levels of SIRT7 in control or SIRT7-KD cells. **E** CCK-8 assays show the growth rates in SIRT7-KD cells with or without CHDL1 restoration. **F** Colony formation assays show colony formation abilities in SIRT7-KD cells with or without CHDL1 restoration. **G**–**I** Colony formation, migration, or sphere formation assays were conducted in the indicated groups. The representative images and quantification data were shown in each assay. **J**–**L** SIRT7 overexpression enhanced the xenograft tumor growth of 786-O cells, and CHD1L KD could abolish this effect. The representative tumor image in the indicated groups is shown on the left (**J**). The tumor volumes were generated (**K**), and tumors were weighed (**L**). *p < 0.05, **p < 0.01, ***p < 0.001, ns no significant

Considering that SIRT7 is less reported in RCC tumorigenesis, we thus intended to focus on the functional relationships between SIRT7 and CHD1L. Firstly, we obtained the expression of SIRT7 from TCGA-KIRC and observed the significantly higher levels of SIRT7 in tumors relative to normal samples. SIRT7 expressions were positively associated with advanced N stages, clinical pathological stages and tumor grades (Additional file 3: Figure S2A). Patients with high SIRT7 expressions have poorer prognosis than those with low SIRT7 levels, as evidenced by Kaplan–Meier analysis in TCGA-KIRC dataset (Fig. 4B). Wild-type SIRT7, but not the defective H187Y mutant, could notably increase growth rates of RCC cells (Fig. 4C). SIRT7 KD notably suppressed cell growth and colony formation capacity, which could be largely rescued by ectopic expression of CHD1L (Fig. 4D–F). In contrast, SIRT7 overexpression could significantly promote colony formation, migration and self-renewal abilities in vitro. The SIRT7-induced RCC malignant features could notably abolished with CHD1L-KD (Fig. 4G–I). To investigate the in vivo role of SIRT7-CHD1L axis in the tumor growth of RCC cells, we utilized the subcutaneous xenograft model. As quantified by the tumor growth curve and tumor weight, we confirmed that SIRT7 could accelerate in vivo tumor proliferation, which could be repressed by CHD1L-KD (Fig. 4J–L). Collectively, these results implicated that high SIRT7 exerted the oncogenic effect during RCC progression depending on CHD1L.

### Accumulated CHD1L mainly modulates HIF-2α-dependent pathway in RCC

**Fig. 5:**
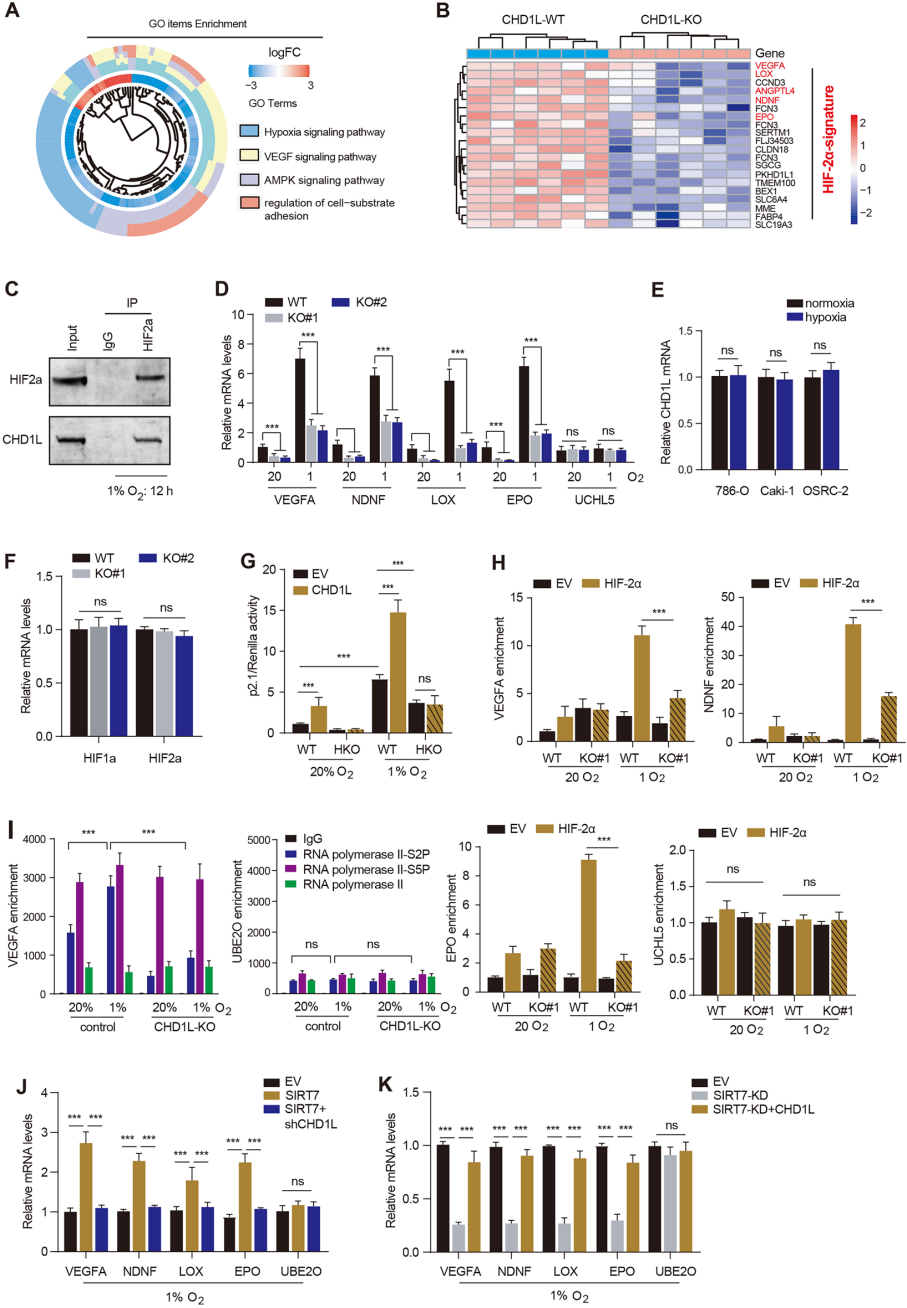
CHD1L binds to HRE of HIF-2α targets to amply this crosstalk under hypoxia. **A** GO enrichment analysis revealed the related biological items based on CHD1L-related genes in the TCGA-KIRC cohort. **B** Heatmap showed the RT-qPCR results of HIF-2α targets in parental and CHD1L-KO 786-O cells. **C** Western blot assay showing the endogenous interactions between CHD1L and HIF-2α. **D** RT-qPCR assays reveal the mRNA levels of HIF-2α targets in control or CHD1L-KD cells under normoxia or hypoxia conditions. **E** RT-qPCR assays reveal the mRNA levels of CHD1L under normoxia or hypoxia. **F** RT-qPCR assays reveal the mRNA levels of HIF-1/2α in control or CHD1L-KO 786-O cells. **G** Parental and HIF-2α-KO 786-O cells were co-transfected with p2.1, PSV-Renilla, and a vector encoding FLAG-CHD1L or EV. Cells were exposed to 20% or 1% O_2_ for 24 h and subjected to dual-luciferase reporter assays (n = 3, mean ± SEM). **H** ChIP-qPCR assays showed the HIF-2α-binding to HREs of genes in parental and CHD1L-KO cells under normoxia or hypoxia for 12 h, individually. **I** RNA polymerase II ChIP-qPCR assays in parental and CHD1L-KO 786-O cells exposed to 20% or 1% O_2_ for 24 h (mean ± SEM, n = 3). **J** RT-qPCR assays showing the mRNA levels of HIF-2α targets of 786-O cells in the indicated groups under hypoxia for 12 h. **K** RT-qPCR assays showing the mRNA levels of HIF-2α targets of 786-O cells in the indicated groups under hypoxia for 12 h. *p < 0.05, **p < 0.01, ***p < 0.001, ns no significant

To further explore the downstream mechanisms that contribute to CHD1L’s oncogenic impact, we calculated and screened the CHDL1-related genes via TCGA-KIRC dataset. A total of 785 CHD1L-related genes were obtained with *Pearson’s* r ≥ 0.32 (Additional file 4: Table S2). Biological enrichment analysis based on CHD1L-related genes showed that hypoxia signaling was highlighted (Fig. 5A). The RT-qPCR assays also confirmed the consistent decrease of hypoxia-related genes in CHD1L-deficient 786-O cells, including VEGFA, LOX, EPO or ANGPTL4 (Fig. 5B). Given that HIF-2α is the master regulator of hypoxia signaling in RCC, we validated the endogenous physical interactions between CHD1L and HIF-2α (Fig. 5C). Although the representative HIF-2α downstream genes (VEGFA, NDNF, LOX, e.g.) were consistently induced by hypoxia, CHD1L-KD could largely ablate these genes expressions (Fig. 5D). However, hypoxia failed to directly regulate CHD1L expressions, and CHD1L did not regulate HIF-1/2 levels (Fig. 5E). We thus speculated that CHD1L may modulate the transcriptional capacity of HIF-2α. To validate this hypothesis, we transfected vector, CHD1L, HIF luciferase reporter (p2.1), pSV-Renilla into 786-O cells and cultured these cells in normoxia or hypoxia for 24 h, individually (Fig. 5F). CHD1L could notably enhance HIF luciferase reporter efficiency under hypoxia, which could be abolished by HIF-2α-KO (HKO) (Fig. 5G). Then, we exposed the control or CHD1L-KO cells to normoxia or hypoxia for 24 h and conducted the ChIP-qPCR assay using anti-HIF-2α antibodies, individually. Indeed, CHD1L deficiency could efficiently destroy the HIF-2α-binding to HREs of targets, especially under the hypoxia condition (Fig. 5H). The non-HIF target UCHL5 was not disturbed. As is previously reported, release of paused RNA polymerase II is indispensible for HIF-2α transactivation [27, 28], during which paused RNA polymerase II is phosphorylated at serine 5 (S5P), and serine 2 phosphorylation (S2P) of RNA polymerase II modulates the release process. We therefore investigated the underlying relationships between CHD1L and paused RNA polymerase II release. We then exposed the parental and CHD1L-loss 786-O cells to normoxia or hypoxia for 24 h, individually. ChIP-qPCR assays found the RNA polymerase II–S2P occupancy on the HIF-2α target (VEGFA) was notably strengthened by hypoxia, and enrichment of RNA polymerase II-S5P was not altered. Only enrichment of RNA polymerase II-S2P, but not the RNA polymerase II-S5P and total RNA polymerase II, was remarkably abolished by CHD1L-KO in hypoxia-treated 786-O cells (Fig. 5I). Occupancy of RNA polymerase II-S2P and others on the non-HIF target UBE2O was not disturbed. Lastly, we also demonstrated that SIRT7 could also depend on CHD1L to activate expressions of HIF-2α targets under hypoxia (Fig. 5J). Although SIRT7-KD could repress the HIF-2α targets, CHD1L could rescue their expressions (Fig. 5K). SIRT7 correlated with HIF-2α targets in TCGA-KIRC cohort (Additional file 3: Figure S2B). Together, these data implicated that CHD1L amplifies HIF-2α transcriptional activity, highlighting novel epigenetic mechanisms underlying HIF transactivation in RCC.

### CHD1L recruits BRD4 to activate HIF-2α-dependent crosstalk and promotes RCC progression in vitro and in vivo

**Fig. 6:**
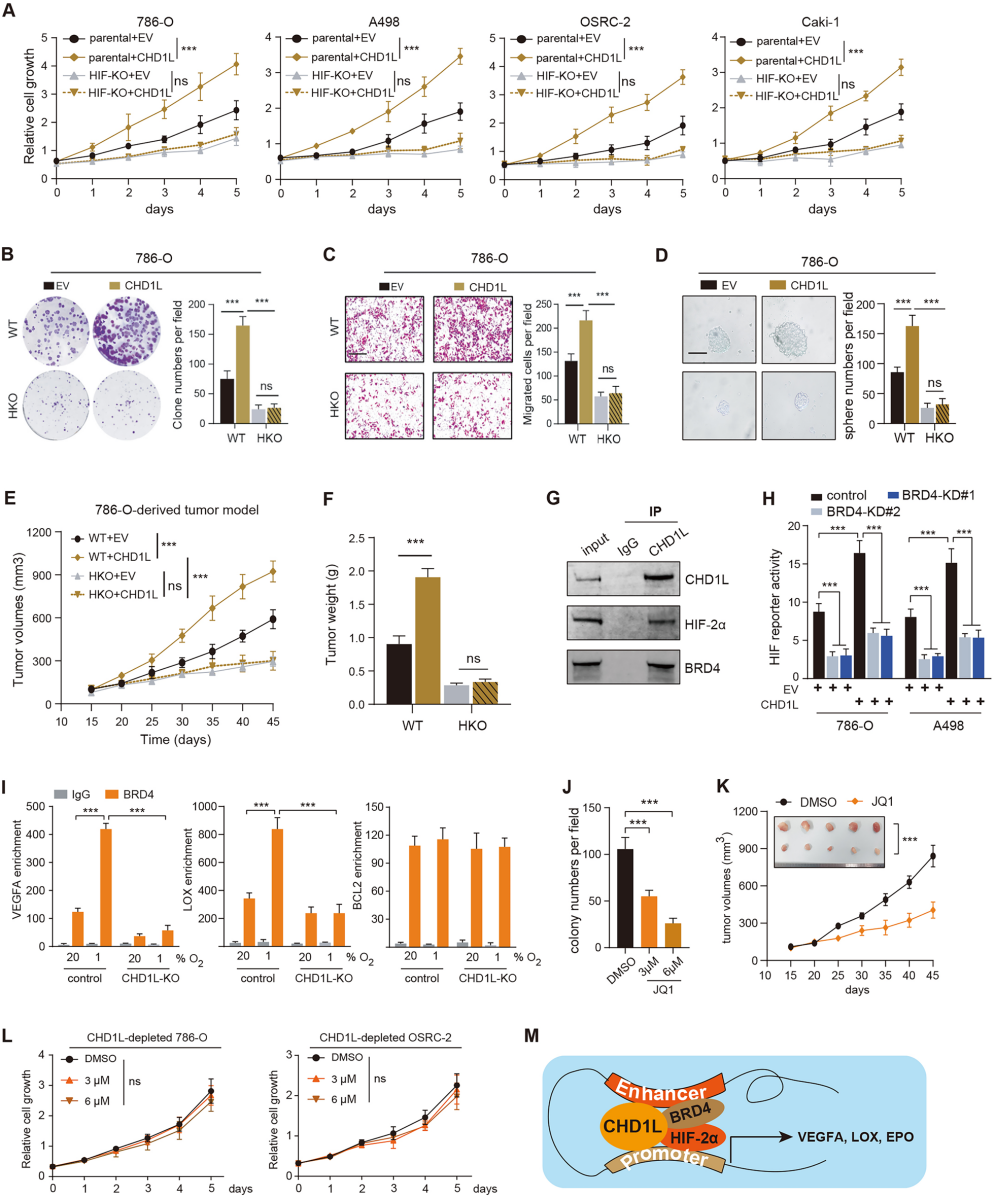
CHD1L enhances colony formation, migration, and stemness of RCC cells depending on HIF-2α. **A** CCK-8 assays showing the cell growth rates in parental and HIF-2α-KO (HKO) 786-O cells expressing EV or CHDL1 exposed to 20% or 1% O_2_ for 12 h. **B**–**D** Colony formation, migration, or stemness abilities were detected in cells in the indicated groups. **E** A subcutaneous tumor model was generated by the indicated cells and the tumor growth curve was shown. **F** Tumor weight was calculated and compared with tumors from the indicated groups. **G** Co-IP assays were used to confirm the endogenous interactions between CHDL1 and BRD4. **H** HIF luciferase reporter assays in 786-O cells transfected with indicated plasmids and exposed to 20% or 1% O_2_ for 24 h in the presence of doxycycline. The FLuc/RLuc activity was determined (mean ± SEM, n = 3). **I** BRD4 ChIP-qPCR assays in parental and CHD1L-KO 786-O cells exposed to 20% or 1% O_2_ for 12 h (mean ± SEM, n = 3). **J** Colony formation assays were conducted in cells treated with increasing doses of JQ1. **K** A subcutaneous tumor model was generated to confirm the in vivo response of RCC cells to JQ1. **L** MTT analysis of CHD1L-KD 786-O and OSRC-2 cells underwent JQ1 treatment with increasing doses (0, 3 μM, or 6 μM). **M** Illustration of CHD1L-hijacked loop with BRD4/HIF-2α. *p < 0.05, **p < 0.01, ***p < 0.001, *ns* no significant

We further investigated that whether HIF-2α-driven pathway is indispensible for CHD1L-induced RCC progression. We generated parental and HKO RCC cells (786-O, A498, OSRC-2, Caki-1) and transduced these cells with lentivirus carrying EV or Flag-CHD1L, individually. The cell growth rates, colony formation capacity, migration, or stemness properties were all enhanced by CHD1L, but HKO abolished the CHD1L-induced RCC progression (Fig. 6A–D). We also implanted these indicated cell lines into the frank of SCID mice, and CHD1L overexpression notably augmented RCC tumor growth in mice. However, HKO completely abolished the CHD1L-induced in vivo RCC growth (Fig. 6E, F). Furthermore, we also found that BRD4 also interacts with CHD1L, and may regulate the CHD1-mediated HIF-2α transactivation (Fig. 6G). BRD4 knockdown could attenuate HIF-2α transcriptional activity, and further abolish the CHD1L-induced HIF-2α activation in hypoxic 786-O or A498 cells (Fig. 6H). ChIP-qPCR assays further confirmed that CHD1L recruits BRD4 to the HREs of the HIF-2α downstream genes. CHD1L deficiency also blocked the hypoxia-induced BRD4 enrichment on the HREs (Fig. 6I). Therefore, targeting BRD4 (JQ1) could also suppress colony formation abilities and in vivo growth of HIF-2α high RCC (Fig. 6J, K). However, JQ1 could not further exert inhibitory effects on the growth of cells when CHD1L was depleted (Fig. 6L). The epigenetic loop containing CHD1L/BRD4/HIF-2α was further elucidated by model diagram (Fig. 6M).

### CHD1L mediates sunitinib resistance and targeting CHD1L is synergistic with sunitinib

**Fig. 7:**
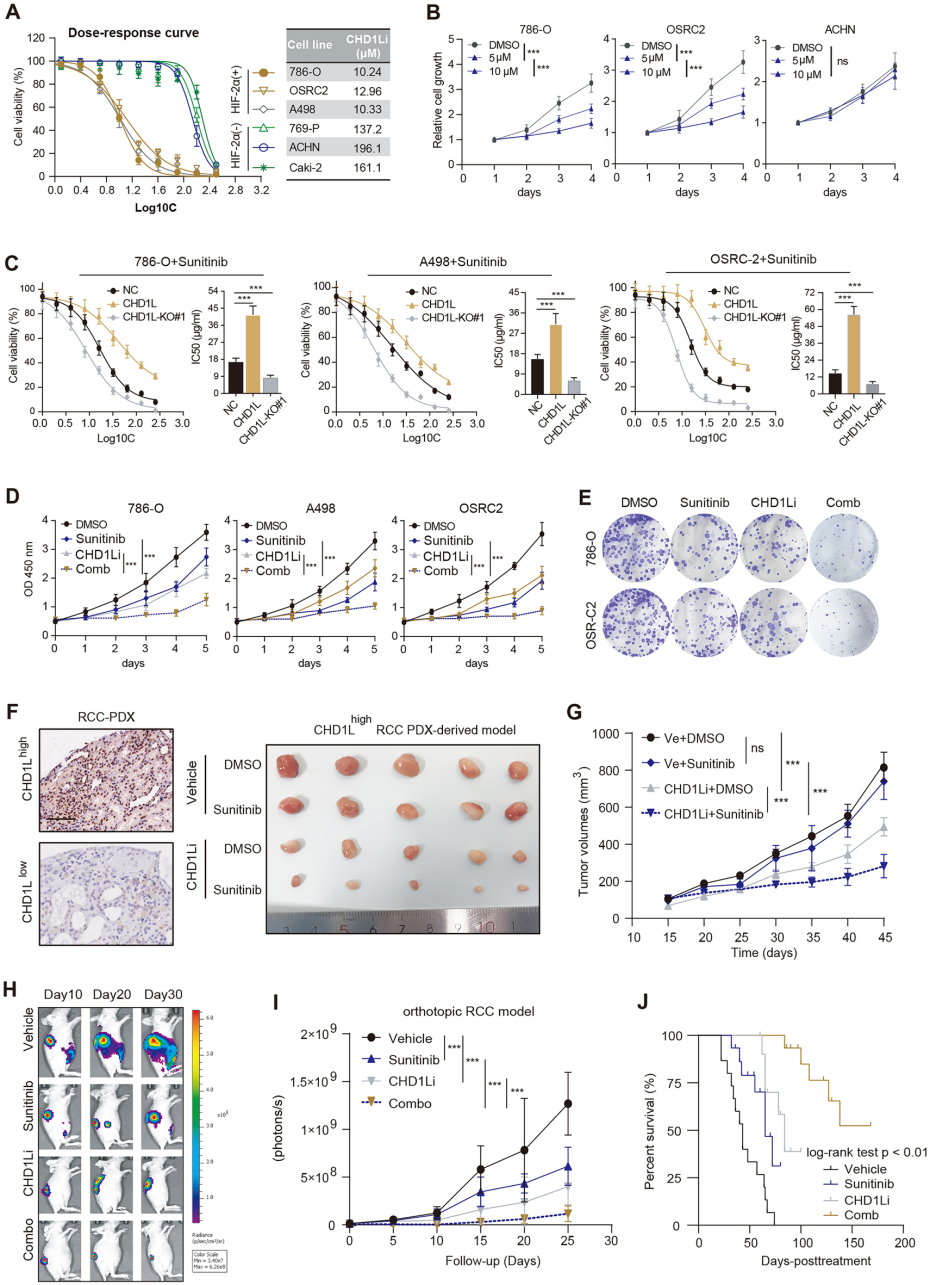
Targeting CHD1L renders HIF-2α^high^ RCC sensitive to sunitinib treatment. **A** Dose–response curves and IC50 of cells treated with CHD1Li. Data are presented as mean ± SD (n = 6) from one-of-three independent experiments. **B** CCK-8 assays showing the efficacy of CHD1Li in suppressing HIF-2α^high^ or HIF-2α^low/−^ RCC. **C** Dose–response curves and IC50 of CHD1L-KO or OE cells treated with sunitinib. **D** CCK-8 assays were used to detect growth rates of cells treated with DMSO, sunitinib, CHD1Li, or Comb. **E** Colony formation assays showed the colony numbers of cells treated with indicated drugs. **F** Immunohistochemistry (IHC) images showed the CHD1L^high^ or CHD1L^low^ RCC-PDXs (left). The subcutaneous tumor model was shown on the right to exhibit tumors from the indicated groups (right). **G** Quantification of the tumor growth curve of tumors from the indicated groups. **H** Representative BLI of orthotopic RCC tumors formed by CMV-Luc 786-O cells in BALB/c nude mice after daily oral gavage with Vehicle control, sunitinib (15 mg/kg), CHD1Li (20 mg/kg), or Comb once daily (n = 8 mice per group). Right: Dynamic measurements of BLI in treated tumors over time. **I** Quantification of BLI signals of mice from the indicated groups. **J** Kaplan–Meier survival curve analysis showed the differential survival outcomes in mice from indicated groups. *p < 0.05, **p < 0.01, ***p < 0.001, ns no significant

**Fig. 8:**
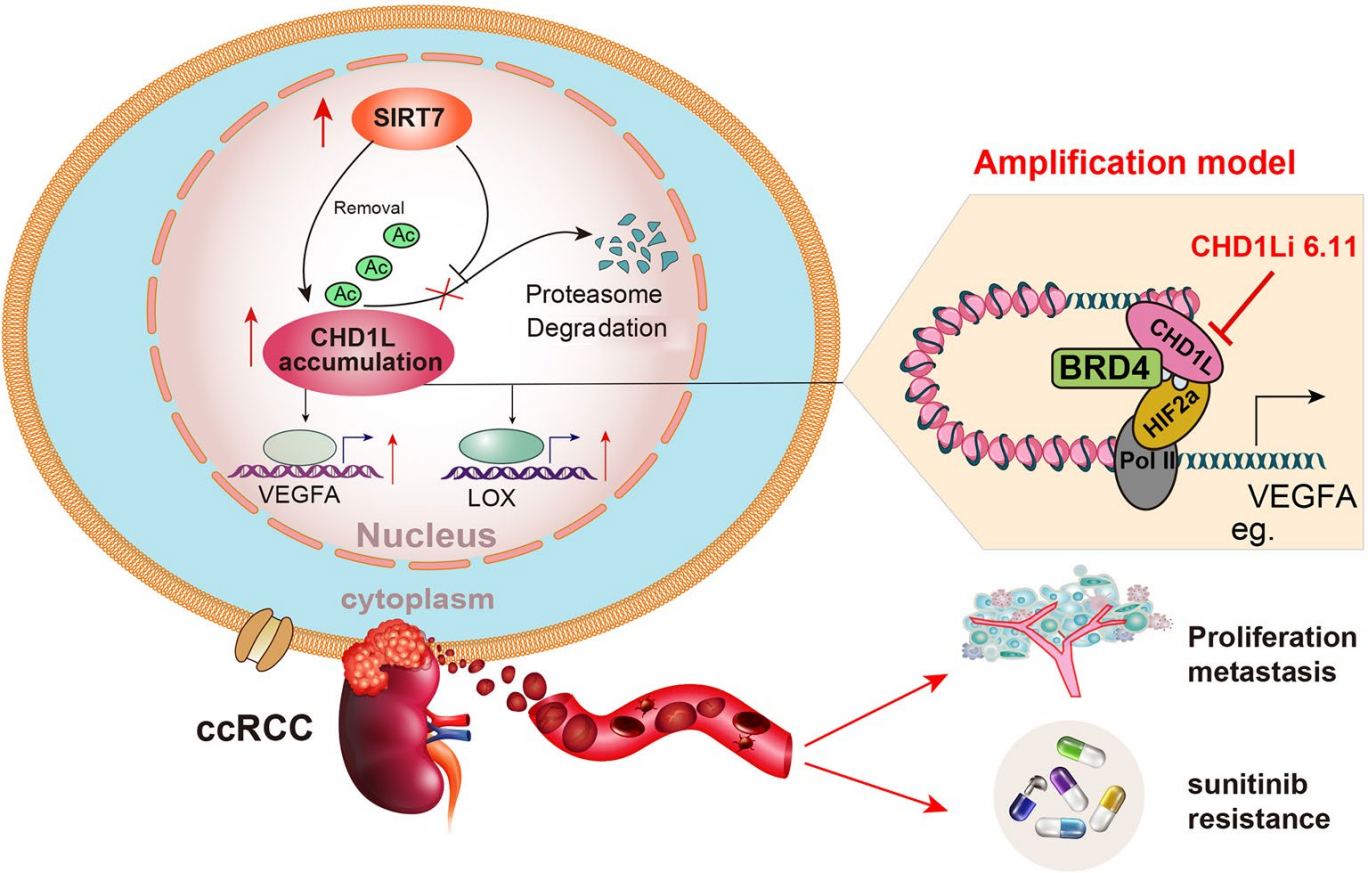
Graphical illustration of SIRT7-CHD1L axis in regulating HIF-2α signaling to promote RCC progression and sunitinib resistance

We also confirmed the pharmacological role of CHD1L inhibitor (CHD1Li 6.11, CHDLi) in suppressing RCC progression [29]. CHD1Li is effective to inhibit HIF-2α-positive RCC, but not the HIF-2α-negative subtype (Fig. 7A). Besides, CHD1Li suppressed HIF-2α-positive RCC (786-O, OSRC-2) in a dose-dependent manner (Fig. 7B). However, CHD1Li markedly suppressed the growth of HIF2^high^ organoids, with only marginal effect in HIF2^low/−^ organoids (Additional file 5: Figure S3A), implicating that targeting CHD1L is effective specifically against HIF2^high^ ccRCCs. CHD1L overexpression mediates the sunitinib resistance, but CHD1L depletion sensitizes RCC to sunitinib (Fig. 7C). CHD1Li has a synergistic effect with sunitinib to suppress cell growth (Fig. 7D, E). In addition, we also generated sunitinib-resistant 786-O cells, defined as 786-O-SR cells. CHD1Li could notably abrogate growth of 786-O-SR cells and further render these cells re-sensitive to sunitinib treatment (Additional file 5: Figure S3B). Given that sunitinib resistance is attributed to the newly identified CHD1L–HIF-2–BRD4 axis, we found JQ1 could also suppress growth of 786-O-SR cells in a dose-dependent manner (Additional file 5: Figure S3C). Patient-derived tumor xenografts (PDXs) were further constructed and categorized into CHD1L^high^ and CHD1L^low^ groups via immunohistochemistry (IHC). CHD1L^high^ RCC showed resistance to sunitinib, but CHD1Li treatment rendered RCC sensitive to sunitinib (Fig. 7F, G). Lastly, luciferase-labeled HIF-2α^high/+^ (786-O) cells were used and orthotopic xenograft was generated in BALB/c mice by subcapsular injection. Combination of CHD1Li and sunitinib exhibited more effectiveness than either one alone (Fig. 7H–J). Collectively, CHD1L is an epigenetic vulnerability for HIF2α^high/+^ RCC. The SIRT7-CHD1L-HIF-2α axis was further illustrated by Fig. 8.

## Discussion

Effective therapeutic strategies suppressing RCC progression are still limited. The intratumor cellular heterogeneity or distinctive molecular characteristics dictates the main challenges [30, 31]. Hypoxia signaling is a well-investigated contributor to RCC that drives tumor growth or metastasis [32, 33]. The aberrant interplay between epigenetic regulators and hypoxia signaling mediates the HIF-2α activation and drug resistance [34, 35]. Here, in this study, we found that CHD1L is up-regulated in RCC and correlates with poorer prognosis of patients. CHD1L enhanced RCC proliferation, migration, and self-renewal capacities in vitro and in vivo. Mechanistically, we explored that CHD1L physically interacts with SIRT7. SIRT7 mediates the deacetylation of CHD1L proteins and thus attenuates its ubiquitination levels. Wild-type SIRT7, but not the defective mutant, prolongs the half-time of CHD1L proteins and stabilizes its levels. SIRT7 relied on CHD1L to accelerate the growth and migration of RCC. When we explored the downstream mechanisms of CHD1L, we observed that CHD1L physically interacts with HIF-2α via binding to the HREs of downstream targets, including VEGFA, LOX, EPO, or NDNF. CHD1L could recruit BRD4 and increase RNA polymerase II phosphorylation at serine 2 (S2P) to promote subsequent transcriptional elongation of the HIF target genes in RCC. CHD1L enhanced HIF-2α transcriptional capacity without altering its levels and positively correlated with its targets in RCC tumors, strongly proving HIF transactivation by CHD1L. CHD1L thus depended on HIF-2α signaling to amplify its downstream oncogenic pathways. Thus, CHD1L is indispensable for HIF-2α^high^ RCC, but not the HIF-2α^low/−^ subtype. CHD1L overexpression promotes sunitinib resistance, and targeting CHD1L could render RCC sensitive to sunitinib treatment. The orthotopi RCC implantation model further confirmed the notable effect of the “Combo” strategy to inhibit RCC via combing CHD1Li and sunitinib.

Previous studies have uncovered several epigenetic regulators that co-activate a series of HIF-2α downstream targets in RCC. For instance, ZMYND8 interacts with HIF-1/2α to enhan elongation of tcehe global HIF-induced oncogenic genes in breast cancer [36]. The p300-mediated ZMYND8 acetylation is indispensable to HIF activation and breast cancer progression and metastasis. Similarly, in this study, we mainly uncovered two aspects of HIF-2α regulation by CHD1L in RCC. First of all, CHD1L enhances the increased HIF-2α binding ability to the HREs of target genes, like VEGFA, LOX, or NDNF. CHDL1 deficiency notably abolished the HIF-2α binding to target genes. Secondly, CHD1L increased the recruitment of RNA Pol II-S2P and BRD4 to promote HIF-2α transcriptional activity. Thus, we found that targeting BRD4 abolished CHD1L-driven HIF-2α downstream activity and notably inhibit HIF-2α^high^ RCC growth in vitro and in vivo. Many Transcriptional Factor (TFs) and chromatin remodeling proteins were found to interact with BRD4. BRD4 could participate in the formation of active enhancers or super-enhancers to amplify local transcriptional capacity. We thus wondered whether BRD4 could amplify the effect of the CHD1L-HIF-2α complex via modulating enhancers. This point is investigated in the following research. RNA polymerase II (Pol II), as the core transcription machine in eukaryotic cells, is mainly responsible for transcribing protein-coding mRNA and some non-coding RNA. Previous studies indicated that RNA polymerase II pausing and release is the pivotal switch for HIF-2α on or off. We observed that CHD1L could notably increase PoII-S2P levels, indicating the release of paused RNA polymerase II. As is well documented, BRD4 interacts with positive transcription elongation factor b (P-TEFb) and activates P-TEFb for RNA polymerase II CTD phosphorylation [37]. We thus speculated that CHD1L may recruit BRD4 to catalyze the P-TEFb complex for Pol II release. However, the underlying regulatory mechanisms need to be further elucidated.

The roles of SIRT7 in tumor development remain to be controversial. In breast cancer, SIRT7 deacetylates and promotes β-TrCP1-mediated SMAD4 degradation. Thus, down-regulated SIRT7 mainly activates transforming growth factor-β signaling and enhances epithelial-to-mesenchymal transition. SIRT7 functions as a tumor suppressor to antagonize breast cancer lung metastasis. Of note, SIRT7-dependent deacetylation could mediate p53 stabilization, which is a famous tumor suppressor [38]. However, SIRT7 reduced the acetylation of MEF2D and expression of PD-L1 in HCC cells to promote HCC cell proliferation [39]. Researchers also discovered the 2800Z and 40569Z compounds to inhibit SIRT7, which highlights novel therapeutic options against liver cancer [40]. Oncogenic SIRT7 could also suppress GATA4 transcriptional activity and activate the Wnt signaling pathway in ovarian cancer [41]. In kidney cancer, we confirmed that SIRT7 is also an oncogenic factor. SIRT7 mediated the deacetylation of CHD1L and impacted its ubiquitination levels. However, the in-depth mechanisms between CHD1L deacetylation and ubiquitination are still unknown. Meanwhile, the roles of SIRT7-mediated histone deacetylation during RCC progression are needed to be further discovered.

We still raised some problems in the current study. Although high SIRT7/CHD1L correlates with a poorer prognosis of RCC, the optimal cutoff that divides patients into high- or low-groups was still unknown. Secondly, CHD1L is only required for HIF-2α^high^ RCC, but not the HIF-2α^low/−^ RCC. Whether HIF-2α inhibitor (PT-2399) is synergistic with CHD1Li remains to be unknown. More pre-clinical models or patient-derived organoids (PDOs) were warranted to confirm the efficacy of CHD1Li in RCC treatment.

## Conclusions

Taken together, the SIRT7-CHD1L-HIF-2α axis is elucidated to be a prognostic axis for predicting RCC prognosis. Our studies further highlighted CHD1L as a potential biomarker and therapeutic target for the diagnosis and treatment of RCC. Targeting CHD1L not only could suppress RCC, but have a synergistic effect with sunitinib.

### Supplementary Information

**Additional file 1: Figure S1.** Raw uncropped western blotting graphs in this study.

**Additional file 2: Table S1.** Clinical information of RCC patients from TCGA-KIRC cohort.

**Additional file 3: Figure S2.** SIRT7 is a prognostic factor in RCC. **(A)** Correlation analysis between SIRT7 and clinical characteristics in TCGA-KIRC cohort. **(B)** SIRT7 levels were associated with HIF-2α targets.

**Additional file 4: Table S2.** Correlation analysis of CHD1L-related genes based on TCGA-KIRC dataset.

**Additional file 5: Figure S3.** Targeting CHD1L sensitizes RCC to sunitinib. **(A)** Representative PDO growth images of HIF2^high^ and HIF2^low/−^ ccRCC PDOs treated with DMSO or CHD1Li (10 μM) for 10 days. Scale bars, 100 μm. **(B)** MTT analysis of 786-O-SR cells treated with DMSO, sunitinib, CHD1Li, or Comb. **(C)** MTT analysis of 786-O-SR cells treated with JQ1. *p < 0.05, **p < 0.01, ***p < 0.001, ns no significant.

## Data Availability

The data used to support the findings of this study are available from the corresponding author upon request.
