# Supplementary figures and images for: The SIRT7-mediated deacetylation of CHD1L amplifies HIF-2α-dependent signal that drives renal cell carcinoma progression and sunitinib resistance
Source: Cell Biosci. 2023 Sep 10;13:166. doi: 10.1186/s13578-023-01113-4 (PMC10493023; doi:10.1186/s13578-023-01113-4)

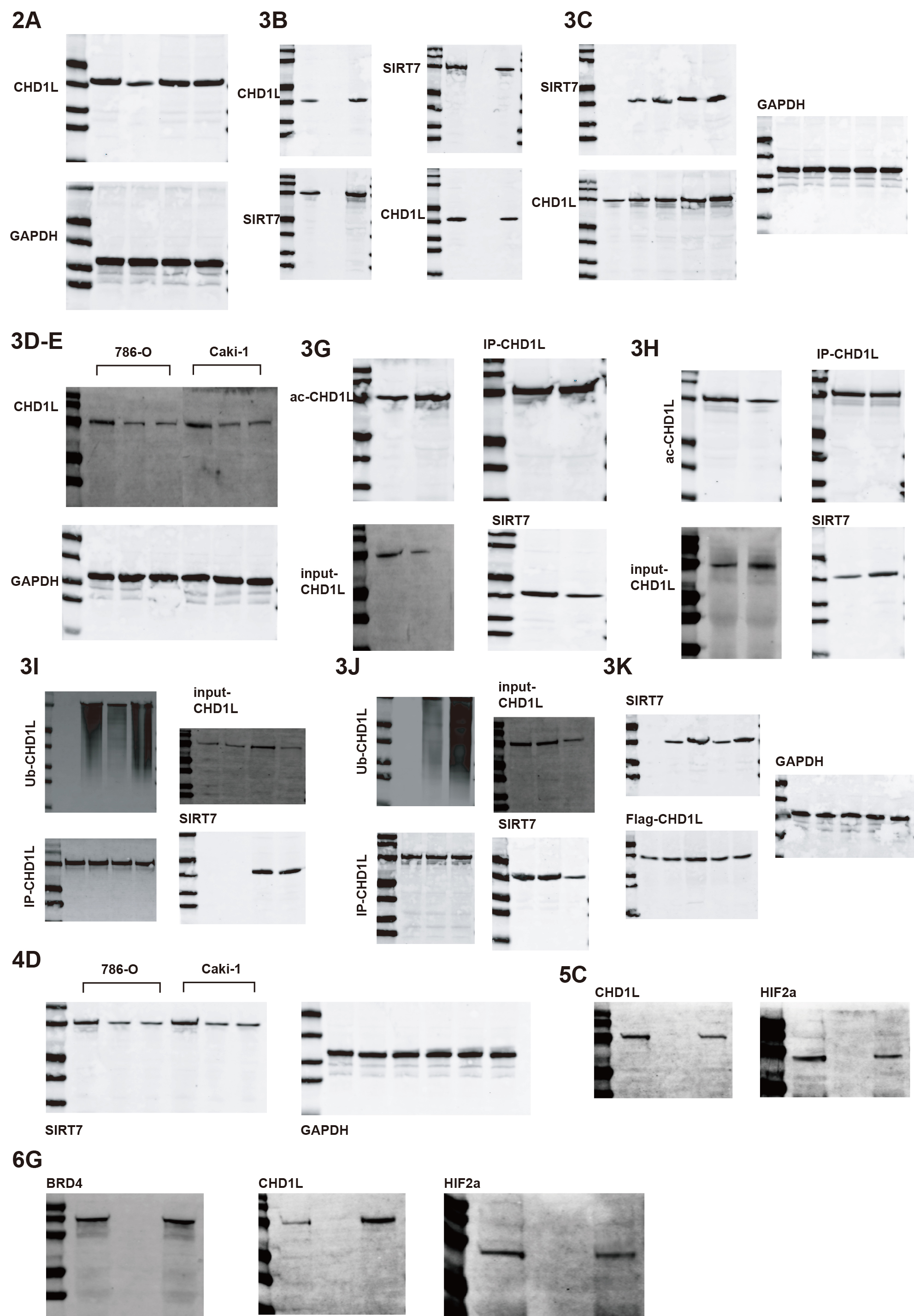

Supplement: Supplementary file 1 — Additional file 1: Figure S1. Raw uncropped western blotting graphs in this study. [file 13578_2023_1113_MOESM1_ESM.jpg]

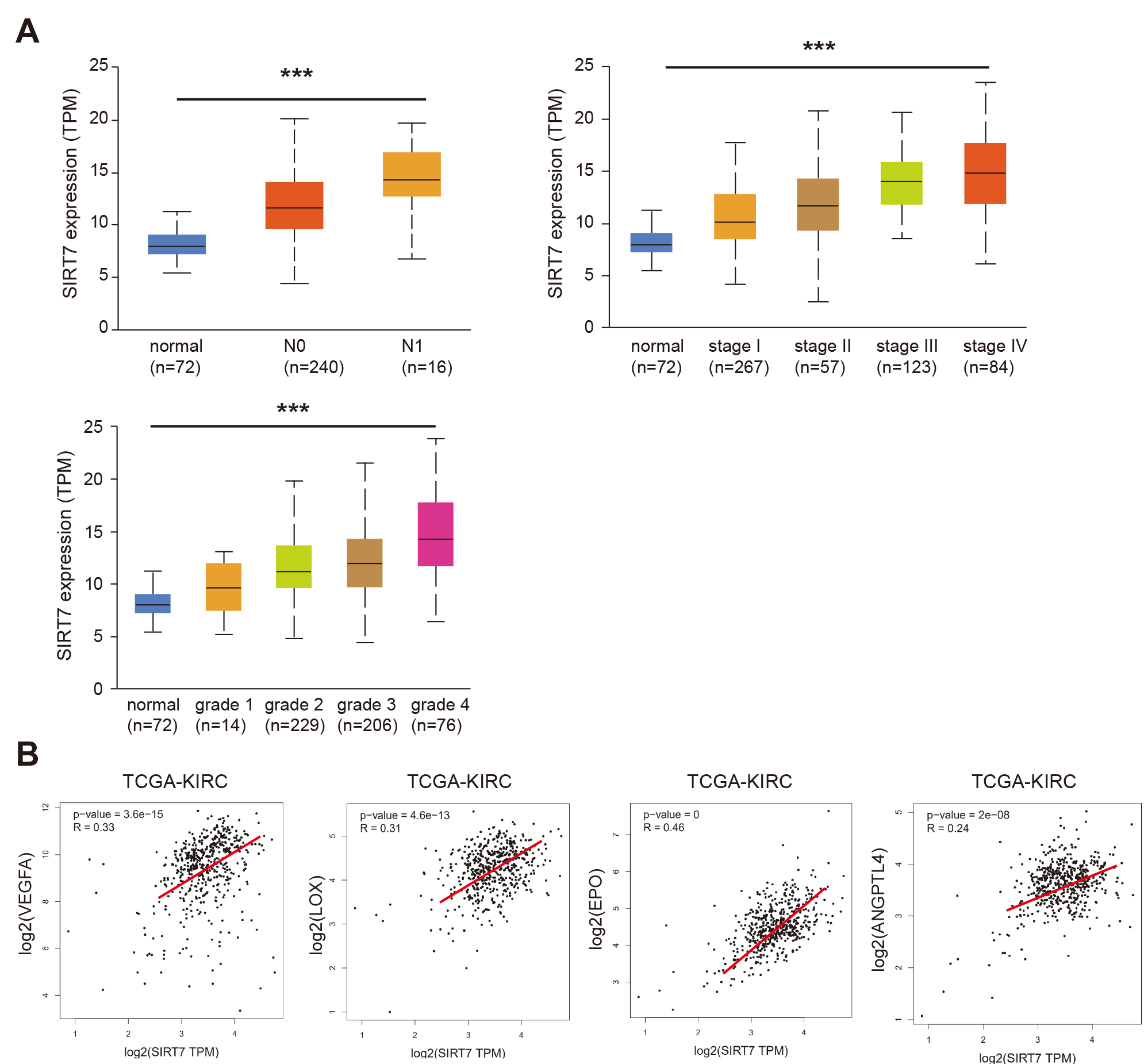

Supplement: Supplementary file 3 — Additional file 3: Figure S2. SIRT7 is a prognostic factor in RCC. (A) Correlation analysis between SIRT7 and clinical characteristics in TCGA-KIRC cohort. (B) SIRT7 levels were associated with HIF-2α targets. [file 13578_2023_1113_MOESM3_ESM.jpg]

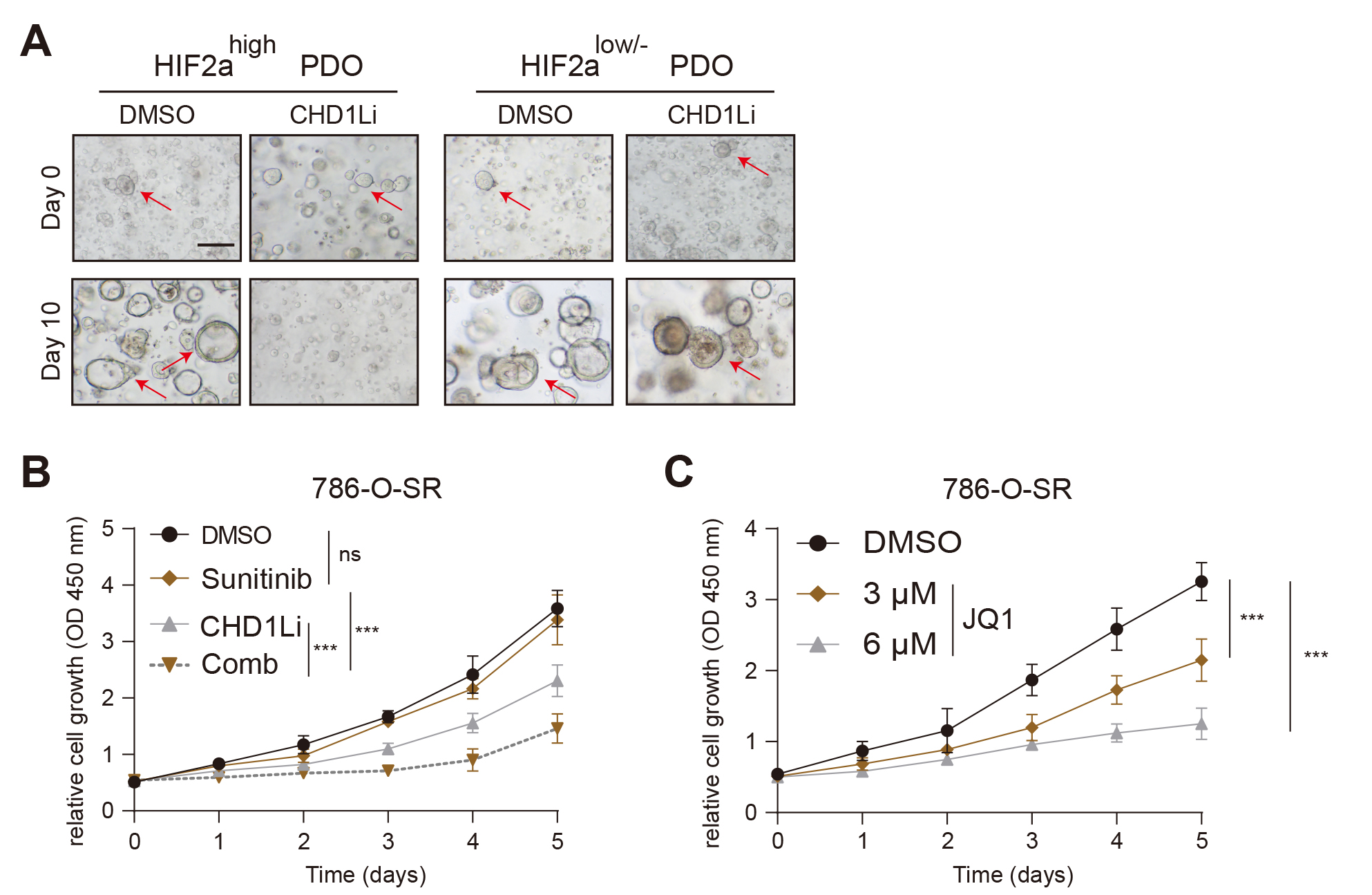

Supplement: Supplementary file 5 — Additional file 5: Figure S3. Targeting CHD1L sensitizes RCC to sunitinib. (A) Representative PDO growth images of HIF2high and HIF2low/− ccRCC PDOs treated with DMSO or CHD1Li (10 μM) for 10 days. Scale bars, 100 μm. (B) MTT analysis of 786-O-SR cells treated with DMSO, sunitinib, CHD1Li, or Comb. (C) MTT analysis of 786-O-SR cells treated with JQ1. *p < 0.05, **p < 0.01, ***p < 0.001, ns no significant. [file 13578_2023_1113_MOESM5_ESM.jpg]
